# Non-invasive biomechanical assessment of the prolapsed vaginal wall: an explorative pilot study on cutometry and indentometry

**DOI:** 10.1038/s41598-023-29403-4

**Published:** 2023-02-16

**Authors:** Yani P. Latul, Arnoud W. Kastelein, Boris C. de Graaf, Zeliha Guler, Jan-Paul W. R. Roovers

**Affiliations:** 1 Department of Obstetrics and Gynecology, Amsterdam UMC location University of Amsterdam, Meibergdreef 9, 1105 AZ Amsterdam, The Netherlands; 2 Amsterdam Reproduction and Development research institute, Amsterdam, The Netherlands; 3 Department of Gynecology, Bergman Clinics, Women’s Health, Amsterdam, The Netherlands

**Keywords:** Physiology, Reproductive biology, Menopause, Urogenital reproductive disorders

## Abstract

The clinical assessment of pelvic organ prolapse (POP) and associated treatment strategies is currently limited to anatomical and subjective outcome measures, which have limited reproducibility and do not include functional properties of vaginal tissue. The objective of our study was to evaluate the feasibility of using cutometry and indentometry for non-invasive biomechanical assessment of the vaginal wall in women with POP. Both techniques were applied on the vaginal wall of 20 women indicated for surgical correction of POP stage two or higher. The primary outcome was the measurement success rate. Measurements were considered successful if biomechanical parameters were generated after a maximum of three attempts. Secondary outcomes included acquisition time, number of attempts to obtain a successful measurement, and biomechanical parameters. Measurements were successfully performed on the anterior vaginal wall of 12 women with cystocele and the posterior vaginal wall of eight women with rectocele. The success rate was 100% for both techniques and acquisition time was under 1 minute for all 20 measurements. Tissue fast elasticity of the posterior vaginal wall (rectocele) was significantly higher than that of the anterior vaginal wall (cystocele) and negatively correlated with age (r = − 0.57, *P* < 0.05). In women with POP, measuring the biomechanical properties of the vaginal wall using cutometry and indentometry is technically feasible. Objective evaluation of biomechanical properties may help to understand the pathophysiology behind surgical outcomes, providing an opportunity for the identification of patients at risk for (recurrent) prolapse, and individualized treatment decisions.

## Introduction

Pelvic organ prolapse (POP), the protrusion of pelvic organs into the vagina, is a prevalent condition that often impairs pelvic floor function (i.e. micturition, defecation and sexual functioning)^1^. Despite the high incidence and well-identified risk factors, little is known about the underlying pathophysiology. According to DeLancey, POP is caused by a combination of the failure of the levator ani muscles and connective tissue attaching the vaginal wall to the pelvis^2^. Biomechanical failure of the vaginal wall itself contributes to the anatomical changes as well^3–6^. Ex vivo studies demonstrated that the vaginal wall of patients with POP has different biomechanical characteristics, such as higher stiffness and lower strength, compared to control patients^5–7^. Vaginal prolapse surgery aims to reconstruct the functional anatomy when conservative strategies fail or are not well tolerated. However, surgical failure rates are high: about 25% of procedures are performed in a patient that has been operated for POP before^8–10^.

To improve surgical outcomes and to develop innovative therapies, it is crucial that vaginal tissue characteristics can be measured and quantified. At present, the clinical assessment of POP is limited to anatomical and subjective outcome measures. Such outcomes do not provide objective information on tissue characteristics or the direct physiological effects of surgery on the vaginal wall. Therefore, methods that objectively and non-invasively quantify vaginal biomechanical characteristics in vivo are urgently needed to better understand and evaluate POP pathophysiology and surgical outcomes.

Cutometry and indentometry are two widely applied methods for non-invasive evaluation of biomechanical properties of soft tissues^11^. Both methods have mainly been applied to the skin within the field of dermatology and cosmetology^12–17^. Cutometry is a suction-based method that has been applied to the vaginal wall before^18,19^. Studies have demonstrated the limited feasibility of different suction-based devices due to their large apertures, which were inappropriate for application on the vaginal wall^18,19^. We hypothesized that a device with a smaller aperture may improve the usability of this method. Indentometry is an indentation-based method that is widely used for ex vivo evaluation of tissues, but has not been applied to the vaginal wall in vivo before. In this explorative pilot study, we evaluated whether cutometry with a small aperture (4 mm) and indentometry enable biomechanical evaluation of the prolapsed vaginal wall. Ultimately, these methods may guide individualized treatment decisions and contribute to the development of innovative treatment modalities and preventative strategies for POP.

## Materials and methods

This explorative observational pilot study was performed in Bergman Clinics Amsterdam, which is the outpatient women’s clinic of the Amsterdam University Medical Centre location Academic Medical Centre (Amsterdam UMC location AMC). The medical ethical committee of the Amsterdam UMC location AMC approved the study protocol under number METC 2018_281 and it was locally approved by the board of directors of Bergman Clinics Amsterdam. All participants received verbal and written explanation of the study procedures and provided written informed consent. All methods were performed in accordance with the relevant guidelines and regulations.

### Measuring instruments

We evaluated two instruments for non-invasive biomechanical assessment of soft tissues: the Cutometer^®^ and the Indentometer^®^, both developed by Courage and Khazaka electronic GmbH, Cologne, Germany. Both instruments were connected to a medical-grade laptop through the Cutometer Dual Multi Probe Adaptor (MPA) 580 system and were operated using the corresponding software, i.e., the Cutometer® dual software and MPA software (Courage and Khazaka electronic GmbH, Cologne, Germany).

#### Cutometer MPA 580

**Figure 1:**
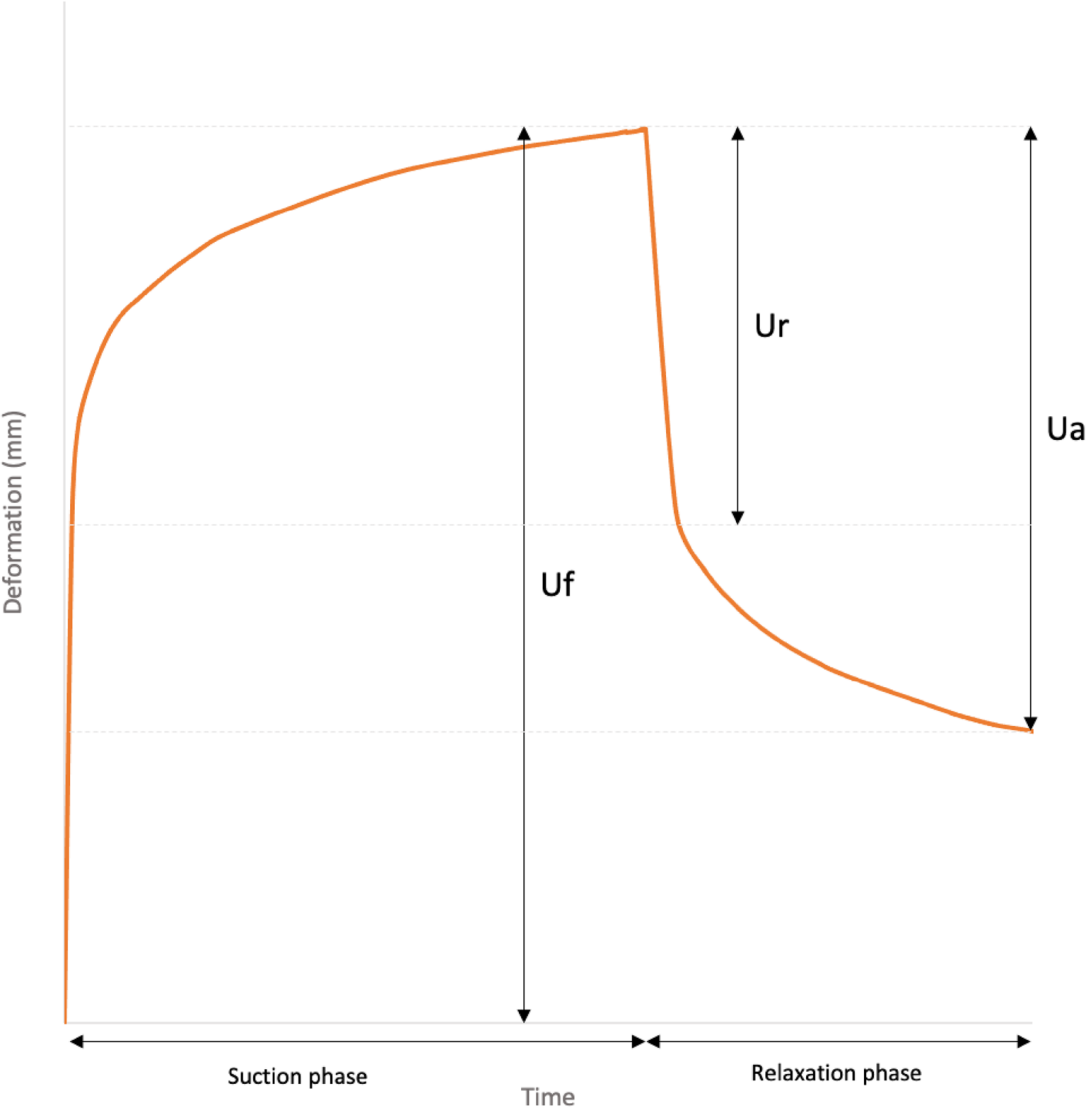
Typical deformation curve obtained with a Cutometer. *Uf* final deformation, measure of tissue firmness, *Ur* immediate retraction, *Ua* total recovery.

The measuring principle of the Cutometer is based on a suction method: following negative pressure, tissue is drawn into to aperture of the probe. The degree of tissue deformation in the probe’s aperture is detected by an optical system inside the probe. After a fixed amount of time, the pressure is released, allowing the tissue to return to its original shape and position. The resistance of the skin to the negative pressure (firmness) and its ability to return to its original position (elasticity) are displayed as curves. From these curves, multiple viscoelastic parameters are automatically calculated, including tissue firmness (Uf = Cutometer parameter R0), tissue total elasticity (total recovery (Ua)/Uf = Cutometer parameter R2) and tissue fast elasticity (immediate retraction (Ur)/Uf = Cutometer parameter R7, Fig. 1). Uf represents the extent of tissue deformation following negative pressure. A higher tissue firmness (Uf) indicates the tissue is more extensible, hence less firm. Tissue total and tissue fast elasticity represent proportions of tissue returning to their original position; the closer these parameters are to 1 (100%), the more elastic the tissue is. The aperture of the Cutometer probe used in this study was 4 mm in diameter. Our measuring protocol comprised three consecutive cycles of suction (negative pressure of 300 millibars during 3 s) and release (3 s).

#### Indentometer IDM 800

The measuring principle of the Indentometer is based on the penetration depth of a small pin (indenter), caused by the constant force of a spring. Immediately after positioning the probe on a surface, the penetration depth of the indenter is displayed on the connected screen. The output consists of an indentation number, which is tissue displacement in mm (0 to 3 mm), representing tissue firmness. With increasing tissue firmness, tissue displacement decreases, resulting in a lower indentation number. The IDM 800 probe used in this study comprised a 3-mm diameter indenter.

### Population and setting

Women with anterior or posterior compartment POP-Q stage ≥ 2 (pelvic organ prolapse quantification (POP-Q) system) scheduled for vaginal prolapse surgery under general anaesthesia were eligible for participation. Baseline characteristics included age, dominant compartment of prolapse and POP-Q stage. We did not perform a sample size calculation but decided on a convenient sample of 20 participants sufficient for the evaluation of feasibility. Consequently, the interpretation of biomechanical parameters has to be done with caution. As part of standard medical care, the patients were accommodated on a surgical table in an operating room kept at a constant temperature of 21 ± 1 °C. After induction of anaesthesia, the patient’s legs were raised in lithotomy position.

### Measurement procedures

Measurements were performed after induction of general anaesthesia, after positioning the patient in lithotomy position and before the start of the surgical procedure. Before each measurement, the calibration of the devices was checked using the specific calibration caps of both instruments. The protruding vaginal wall was visualized by manually spreading the labia and subsequently cleared from mucus. Measurements were performed in the midline of the protruding vaginal wall (i.e. anterior vaginal wall for cystocele and posterior vaginal wall for rectocele) at a standardized location of 3 cm from the introitus. First, we performed measurements using the Indentometer by gently placing the probe on the protruding vaginal wall at a 90-degree angle. As recommended by the manufacturer, we performed three consecutive measurements and calculated their mean. If one of the Indentometer measurements failed, we continued our attempts to obtain a total of three successful measurements and noted the number of failed attempts. Subsequently, we performed measurements using the Cutometer on macroscopically the same position as the Indentometer measurement. The probe of the Cutometer was gently placed on the protruding vaginal wall at a 90-degree angle and the measuring protocol was started using the Cutometer dual software. If the first Cutometer measurement failed, a second or even third attempt was made to obtain a successful measurement. Hence only one round containing three cycles of suction and release was obtained per patient. All measurements were performed by the same investigator.

### Outcomes

The primary outcome was the success rate of both techniques. Cutometry was considered successful when application resulted in a deformation curve from which tissue firmness (Uf), total elasticity (Ua/Uf) and fast elasticity (Ur/Uf) could be determined after a maximum of three attempts. Indentometry was considered successful if an indentation number (tissue displacement in mm) could be generated after a maximum of three attempts. Secondary outcomes were determined to provide more insight into feasibility and practicability of both methods and included acquisition time and the number of attempts until successful measurements. Additionally, differences in biomechanical parameters between cystocele and rectocele were assessed to evaluate the ability of the Cutometer and Indentometer to distinguish between groups. Differences between POP stages and correlations between biomechanical parameters and baseline characteristics were evaluated grouped and for the cystocele and rectocele groups separately.

### Statistical analysis

All analyses were performed using IBM SPSS Statistics (IBM Corp. Released 2020. IBM SPSS Statistics for Macintosh, Version 27.0. Armonk, NY: IBM Corp). Continuous data were tested for normality using the Kolmogorov–Smirnov test. Normally distributed continuous variables are presented as means and standard deviations (SD), non-normally distributed continuous variables are presented as medians (μ) and interquartile ranges (IQR) and categorical variables are presented as absolute and relative frequencies.

Between-group comparative analysis was performed using the independent samples *t* test for normally distributed continuous data, Mann–Whitney *U*-test for non-normally distributed continuous data, and Pearson’s Chi-squared test for categorical data. Correlations between biomechanical parameters and age were evaluated by calculating the Pearson correlation coefficient for normally distributed variables and the Spearman correlation coefficient for non-normally distributed data. A two-sided p-value below 0.05 was considered statistically significant.

## Results

### Patient and measurement characteristics

Measurements were performed on the protruding vaginal wall of 20 women with a mean age of 58.0 ± 14.3 years. Of these, 12 had anterior and 8 had posterior compartment prolapse. Baseline characteristics were not significantly different between women with anterior and posterior compartment prolapse (Table 1). Measurements were performed on 12 anterior and 8 posterior vaginal walls. The criteria for successful measurement were met by all individual measurements using both instruments. All Cutometer measurements were successful on the first attempt. Two of the 20 measurements using the Indentometer failed in the first and second attempts, meaning that no indentation number was generated. In both cases, the third attempt was successful. With respect to secondary outcomes, acquisition time was under 1 min in all measurements and decreased with increasing experience. Measurements with both instruments were convenient, easy to perform and did not damage vaginal tissue.

**Table 1:** Patient characteristics and biomechanical parameters obtained with the Cutometer and Indentometer.

Patient characteristics	Total (n = 20)	Cystocele (n = 12)	Rectocele (n = 8)	*P* value*
Age (years)^a^	58.3 ± 14.3	61.1 ± 14.8	54.1 ± 13.3	0.30
POP stage 2, N (column %)^b^	13 (65%)	6 (50%)	7 (88%)	0.16
POP stage 3, N (column %)^b^	7 (35%)	6 (50%)	1 (13%)	0.16

Biomechanical parameters	Total (n = 20)	AVW (n = 12)	PVW (n = 8)	*P* value**
Tissue firmness (Uf)^a^	1.27 ± 0.29	1.30 ± 0.34	1.23 ± 0.20	0.62
Tissue total elasticity (Ua/Uf)^a^	0.73 ± 0.09	0.71 ± 0.08	0.77 ± 0.10	0.10
Tissue fast elasticity (Ur/Uf)^a^	0.45 ± 0.13	0.40 ± 0.10	0.53 ± 0.15	** < 0.05**
Indentation number^c^	2.78(2.47–2.95)	2.83(2.49–2.95)	2.59(2.47–2.95)	0.73

a unpaired *t* test.

b Pearson chi-square test.

c Mann–Whitney-*U* test.

Bold *P* values indicate statistical significance (i.e., *P* < 0.05).

*Comparison between cystocele and rectocele.

**Comparison between AVW (anterior vaginal wall) from cystocele patients and PVW (posterior vaginal wall) from rectocele patients.

### Biomechanical parameters

**Figure 2:**
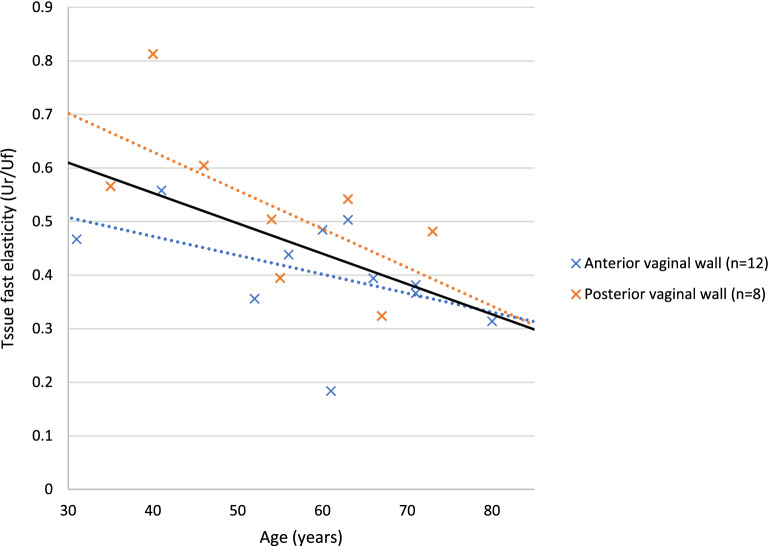
Scatterplot demonstrating the correlation between age and tissue fast elasticity (Ur/Uf) of the anterior (blue, n = 12) and posterior (orange, n = 8) vaginal wall. The continuous black trendline demonstrates the strong negative correlation of all measurements combined (n = 20, r = − 0,57, *P* < 0,05)*.* Dotted trendlines demonstrate the correlation of the anterior (orange, r = − 0,47, *P* = 0,12) and posterior (blue, r = -0,65, *P* = 0,08) vaginal wall separately.

Tissue fast elasticity (Ur/Uf) of in the posterior vaginal wall was significantly higher than of the anterior vaginal wall (Table 1). No statistical differences in tissue firmness (Uf), tissue total elasticity (Ua/Uf) and indentation number were observed between the anterior and posterior vaginal wall (Table 1). Biomechanical parameters were not significantly different between POP stages in patients with cystocele, nor in patients with rectocele (Table 2). Age and tissue fast elasticity (Ur/Uf) demonstrated a strong negative linear correlation; i.e., with increasing age, tissue fast elasticity significantly decreased (Fig. 2). No correlation between age and the other biomechanical parameters was observed. POP-stage did not correlate with any of the biomechanical parameters. No correlation between indentation number and tissue firmness could be demonstrated.

**Table 2:** Comparison of biomechanical parameters between POP stages, separate for the anterior and posterior vaginal wall.

Anterior vaginal wall (cystocele, n = 12)	POP 2 (n = 6)	POP 3 (n = 6)	*P* value
Tissue firmness (Uf) ^a^	1.28 ± 0.28	1.32 ± 0.42	0.13
Tissue total elasticity (Ua/Uf)^a^	0.67 ± 0.08	0.74 ± 0.06	0.52
Tissue fast elasticity (Ur/Uf)^a^	0.37 ± 0.12	0.43 ± 0.07	0.73
Indentation number^b^	2.83 (2.28–2.92)	2.85 (2.60–2.95)	0.46

Posterior vaginal wall (rectocele, n = 8)	POP 2 (n = 7)	POP 3 (n = 1)	*P* value
Tissue firmness (Uf)^a^	1.24 ± 0.22	1.19	*-*
Tissue total elasticity (Ua/Uf)^a^	0.77 ± 0.11	0.83	*-*
Tissue fast elasticity (Ur/Uf)^a^	0.53 ± 0.16	0.54	*-*
Indentation number^b^	2.61 (2.52–2.95)	2.45 (2.45–2.45)	0.26

a unpaired *t* test.

b Mann–Whitney-*U* test.

*POP* pelvic organ prolapse.

Bold *P* values indicate statistical significance (i.e., *P* < 0.05).

## Discussion

### Main findings

This study shows that cutometry and indentometry enable non-invasive biomechanical assessment of the vaginal wall in women with POP. We demonstrated that both methods successfully and rapidly generate objective, quantitative biomechanical parameters. Our results suggest that fast elasticity differs between the anterior and posterior vaginal wall and decreases with increasing age.

### Interpretation of results

The Cutometer and comparable suction-based devices have been applied to the vaginal wall before^18–25^. Epstein et al.^20^ used the Dermalab skin probe (Cortex Technology, Hadsund, Denmark) to investigate the stiffness of the vaginal wall in patients with POP, its relation to the severity of symptoms and the effect of sacral colpopexy^20–22^. Webrouck et al. (2010) have evaluated the feasibility of a Cutometer device with 6- and 8-mm aperture for the assessment of vaginal biomechanical properties and demonstrated that measurements within the same patient, at different sites, showed a positive correlation^18,19^. In turn, two studies reported on another suction-based device, the BTC-2000™^23,24^. Mosier et al. (2011) demonstrated good intra- and inter-rater reliability of the device for the application to the vaginal wall of women with POP^23^, and Chuong et al. (2014) demonstrated that the prolapsed anterior vaginal wall of patients with POP was more compliant than the suprapubic skin^24^. Finally, Röhrnbauwer et al. (2017) used a probe called “the aspiration device” (Fiberoptic P. + P. AG, Spreitenbach, Switzerland), which contains a lateral aperture, facilitating easy intravaginal application^25^.

In summary, multiple suction-based devices have been applied to the vaginal wall to measure biomechanical characteristics in vivo. All devices have different properties, settings and outcome measures, (stiffness (index), elasticity, extensibility, compliance, tissue displacement). Therefore, we cannot directly compare the reported outcomes of these studies. Some authors described significant biomechanical differences between women with and without POP, whilst others did not. One of the main issues of the devices used seems to be the rather large apertures of 10 mm to 13.5 mm. Such large apertures are inappropriate for measurements of the vaginal wall, as tissue may enter the aperture even before negative pressure is built up because of its compliance. Therefore, in our study, we used a device with a much smaller aperture of 4 mm. Theoretically, this could enable more accurate assessment, as tissue deformation actually results from the negative pressure inside the probe, and for example not from the pressure that is applied to position the probe. However, a smaller aperture also implies that less tissue is aspirated into the aperture and therefore, only the superficial layers of tissue are assessed. Directly comparing different apertures and the effect of the aperture on measuring depth should be a subject of future research.

The Indentometer and similar instruments have not been applied to the vaginal wall before. Compared to the reported indentation numbers of the skin, our results demonstrate higher indentation numbers, indicating lower vaginal tissue firmness than the skin^26,27^. This is in line with our observation that vaginal tissue firmness—obtained with the Cutometer—was lower (higher Uf) than dermal tissue firmness described in the literature, but we could not demonstrate a significant correlation between the two parameters. The indentation number generated by the Indentometer ranges from 0 to 3 mm. Overall, we observed indentation numbers of the vaginal wall that are close to the maximum value, indicating that the firmness of the vaginal wall is relatively low for the detectable range.

### Strengths and limitations

We would like to point out some strengths of our study. First, we introduced two non-invasive methods to measure vaginal tissue biomechanics in vivo. We demonstrated that the use of these methods is feasible for vaginal measurements, and can distinguish between different groups with different biomechanical characteristics. This creates enormous potential for future research that focuses on an improved understanding of pelvic organ prolapse and the evaluation of newly designed surgical and non-surgical treatment modalities. All measurements were performed by a single researcher to prevent interobserver variability. However, some limitations and uncertainties need to be addressed as well. First, as the primary objective of this study was to evaluate the feasibility of the devices, secondary outcomes need to be interpreted with caution. The study was not designed to evaluate biomechanical parameters; therefore, we should not draw firm conclusions based on these results. Future studies need to evaluate the reproducibility and reliability of measurements before we can accurately interpret the biomechanical parameters that the devices produce. Second, there are several potential influences on biomechanical parameters that have not been assessed in the current study, i.e., the roughness of the vaginal wall (vaginal rugae), active muscle contraction, the effect of anaesthesia and filling of the bladder and rectum. Third, in their current form, the probes of both techniques must be held in a perpendicular position to the tissue of interest, making them unsuited for intravaginal application and therefore unsuited for the assessment of patients without POP or with reconstructed POP. For the Cutometer, this limitation has been reported before^17^. Therefore, in this study, we performed measurements on the accessible, protruding vaginal wall of patients with POP to or beyond the hymen. Consequently, we could not compare our findings to a control group without POP, or following surgical correction. Therefore, it remains unclear to what extent the obtained biomechanical properties differentiate physiology from pathology.

### Conclusion and future perspectives

This study introduces two feasible alternative approaches for the evaluation of vaginal tissue biomechanics, with the potential to be beneficial in both clinical and research settings. These techniques generate functional rather than anatomical outcome measures, are objective, quantitative and reproducible and are non-invasive and easy to use. Both techniques provide an opportunity for longitudinal, objective evaluation of vaginal tissue characteristics, which may enable clinicians and researchers to evaluate patient-specific tissue characteristics and treatment effects and identify affected women most likely to benefit from synthetic mesh or native tissue surgical repair. Non-invasive biomechanical evaluation of vaginal tissues has the potential to be broadly applicable in the field of gynaecology, in the assessment of other vaginal pathological conditions such as vaginal atrophy and birth trauma. Future research should evaluate whether biomechanical properties correlate to ex vivo tensile testing and connective tissue composition in explants. Furthermore, it should be investigated whether biomechanical parameters correlate to anatomical assessment and subjective symptoms, and whether they provide insight into the chances of success of native tissue repair and other treatment strategies. Ultimately, this may personalize the treatment for the individual patient with POP with higher chances of success and lower failure rates.

## Data Availability

The dataset generated during the current study is freely available in DANS EASY data repository through https://easy.dans.knaw.nl/ui/datasets/id/easy-dataset:254856.
